# A case study of petrophysical rock typing and permeability prediction using machine learning in a heterogenous carbonate reservoir in Iran

**DOI:** 10.1038/s41598-022-08575-5

**Published:** 2022-03-16

**Authors:** Erfan Mohammadian, Mahdi Kheirollahi, Bo Liu, Mehdi Ostadhassan, Maziyar Sabet

**Affiliations:** 1 Key Laboratory of Continental Shale Hydrocarbon Accumulation and Efficient Development, Ministry of Education, Northeast Petroleum University, Daqing, 163318 China; 2 School of Mining Engineering, College of Engineering, University of Tehran, Tehran, Iran; 3 Petroleum and Chemical Engineering, Universiti Teknologi Brunei, Bandar Seri Begawan, Brunei Darussalam

**Keywords:** Environmental sciences, Solid Earth sciences

## Abstract

Petrophysical rock typing (PRT) and permeability prediction are of great significance for various disciplines of oil and gas industry. This study offers a novel, explainable data-driven approach to enhance the accuracy of petrophysical rock typing via a combination of supervised and unsupervised machine learning methods. 128 core data, including porosity, permeability, connate water saturation (S_wc_), and radius of pore throats at 35% mercury injection (R_35_) were obtained from a heterogeneous carbonate reservoir in Iran and used to train a supervised machine learning algorithm called Extreme Gradient Boosting (XGB). The algorithm output was a modified formation zone index (FZIM*), which was used to accurately estimate permeability (R^2^ = 0.97) and R_35_ (R^2^ = 0.95). Moreover, FZIM* was combined with an unsupervised machine learning algorithm (K-means clustering) to find the optimum number of PRTs. 4 petrophysical rock types (PRTs) were identified via this method, and the range of their properties was discussed. Lastly, shapely values and parameter importance analysis were conducted to explain the correlation between each input parameter and the output and the contribution of each parameter on the value of FZIM*. Permeability and R_35_ were found to be most influential parameters, where S_wc_ had the lowest impact on FZIM*.

## Introduction

Rock typing can be defined as dividing the reservoir into zones with identical petrophysical and flow characteristics^1–3^. Rock typing can be used in various industry disciplines, including finding thief zones in drilling, managing zones with high productivity index in the production phase, detecting zone of interest and building robust numerical reservoir models^4–6^. One of the most important usages of rock typing is predicting unknown reservoir properties, specifically permeability in uncored intervals^7^. Coring from several wells is an often unavoidable, essential task in order to obtain basic data on the field. However, coring in all wells of huge fields or from all zones of interest in a single well poses a substantial financial burden. Therefore, rock typing can be used as a method to alleviate these excessive expenses^8^.

Several approaches have been proposed in order to perform appropriate rock typing. Some methods are based on geological aspects of reservoirs such as the Lucia method^9^. Moreover, different petrophysical methods have been defined according to some reservoir properties such as porosity (φ), permeability (K), and capillary pressure (Pc). In addition, various empirical and also theoretical indices have been introduced for petrophysical rock typing (PRT), and the most practical ones are summarized in Table1. Rocks in each specific rock type have similar static (Pc, S_wc_, etc.) or dynamic (related to fluid flow behavior) i.e., Petrophysical Static Rock Typing (PSRT) and Petrophysical dynamic Rock Typing PDRT, respectively^10^. In the literature, the terms petrophysical rock types (PRTs) and Hydraulic flow units (HFU) have been interchangeably used. Kadkhodaie 2018 comprehensively reviewed various rock typing methods used in the literature and industry^11^. The formation zone index (FZI), which was a modification of Kozeny–Carman equation, expresses the correlation among micro-scale parameters like pore shape and size, pore throat radius, and aspect ratio to porosity and permeability as shown in Eq. (1), where ${r}_{mh}$ is the average radius of the hydraulic unit, ${F}_{s}$ is the shape factor, and $\tau$ is the hydraulic tortuosity (the ratio of actual to the straight length ($\frac{{L}_{a}}{L}$)). The correlation between these micro-scale attributes and macro-scale easy-to-measure parameters (i.e., porosity and permeability from RCAL) can be made by theoretical studies^12^. To calculate FZI using this method, Reservoir Quality Index (RQI) and normalized porosity (φz) need to be calculated using Eqs. (2) and (3). And finally, Eq. (4) can be used to calculate FZI.

**Table 1:** The most common indices for rock typing.

Authors	Type	Index
Winland^18^	Empirical	$Log\left({R}_{35}\right)=-0.996+0.588Log\left(k\right)-0.864Log(\varnothing )$
Kolodzie^18^	Empirical	$Log\left({R}_{35}\right)=-0.9008+0.554Log\left(k\right)-0.903Log(\varnothing )$
Pittman^19^	Empirical	$Log\left({R}_{20}\right)=-0.388+0.519Log\left(k\right)-0.303Log(\varnothing )$ And $Log\left({R}_{25}\right)=-0.496+0.531Log\left(k\right)-0.350Log(\varnothing )$
Amaefule et al.^20^	Theoretical	$FZI=\frac{0.0314\sqrt{\frac{k}{\varnothing }}}{\frac{\varnothing }{1-\varnothing }}$
Nooruddin and Hossain^13^	Theoretical	$FZIM=\frac{0.0314\sqrt{\frac{k}{\varnothing }}}{\frac{{\varnothing }^{m}}{1-\varnothing }}$
Izadi and Ghalambor^14^	Theoretical	$MFZI=\frac{0.0314\sqrt{\frac{k}{\varnothing }}\times \sqrt{1-{S}_{WC}}}{\frac{\varnothing }{1-\varnothing }{(1-{S}_{WC})}^{2}}$
Mirzaei-Paiaman et al.^21^	Theoretical	${FZI}^{*}=0.0314\sqrt{\frac{k}{\varnothing }}$
Mirzaei-Paiaman et al.^21^	Theoretical	$PSRTI=0.0314\sqrt{\frac{k}{\varnothing }{F}_{S}\tau }$
Mirzaei-Paiaman et al.^15^	Theoretical	$TEM=\frac{k{k}_{r}}{\varnothing \mu }$

1 $$k=\varnothing \frac{{{r}_{mh}}^{2}}{{F}_{s}\tau },$$

2 $$RQI=0.0314\sqrt{\frac{k}{\varphi }},$$

3 $${\varphi }_{z}=\frac{\varnothing }{1-\varnothing },$$

4 $$FZI=\frac{RQI}{{\varphi }_{z}}.$$

Several researchers have tried to modify FZI by the inclusion or by reducing the number of parameters required to calculate the index^13–16^. One of the most critical applications of rock typing is to predict unknown reservoir properties for parts of formation where costly core samples are not available. Hence, various theoretical and empirical models are proposed by which different properties, most important of which is K and porosity (φ), can be estimated. Table 1 shows some theoretical and empirical indices commonly used for rock typing in the literature. In addition to FZI, Winland empirical equation, which relies upon pore throat radius when mercury saturation is 35%, is also a widely used index for rock typing. More recently, Mirzami-Piaman et al. proposed FZI* and TEM that rely on routine core analysis (RCAL) and special core analysis (SCAL)-driven properties such as viscosity and relative permeability of phases^15,17^.

Artificial intelligence (AI) is becoming an essential part of the engineering toolkit in recent years. It has been used to solve a range of problems including mobile computing^22^, solar radiation estimation^23^, assessment of discharge rate of streamflows^24^, forecasting weather^25,26^, and aquacultural engineering^27^. The use of AI, specifically data-driven/machine-learning (ML) algorithms, has gained much attention in the energy, oil, and gas industry^28^. These methods are generally used to estimate the number of parameters that are otherwise technically or theoretically challenging to obtain^29,30^. The data-driven approaches have been used in various disciplines of the industry, such as approximation of multiple parameters related to carbon capture and sequestration^31,32^, predicting oil recovery^33^. And estimation of rock and fluid properties^32,34–36^.

Recently, data-driven strategies have been used for rock typing/clustering of the reservoirs as well. Table 2 summarizes the application of different data-driven algorithms for rock typing purposes. Most previous studies used a variation of support vector machine (SVM) and artificial neural network (ANN) to classify the reservoir into homogenous clusters. Moreover, the majority of previous studies rely upon a combination of core data and well-logging/seismic data as inputs in their models^37^. Additionally, most of the previous researchers disregarded S_wc_ in their indices due to the added complexity to the theoretical model or making the empirical results challenging to interpret (due to the high distribution of values for each rock type compared to porosity and permeability)^38^. S_wc_ is known to play a crucial role in fluid flow in porous media due to its effect on the relative permeability of fluid Hence, omitting it from the equation may result in suboptimal rock types. Hence, in this research, a novel data-driven model (a combination of optimized supervised and unsupervised machine learning methods) that relies on core-data is utilized to propose a modified FZI number (FZIM*) that in addition to the basic petrophysical properties (K and φ) includes S_wc_ and R_35_, both of which is proven to be of significance^17^. In this study, by using FZIM*, the heterogeneous carbonate reservoir of interest is classified into different PRTs. The developed model is explainable from statistics and petroleum engineering points of view. In addition, by using this approach, reservoir parameters such as K and R_35_ can be estimated for uncored parts of the formation.

**Table 2:** Application of machine learning algorithms in reservoir studying.

Authors	Applied algorithm	Input parameters	Prediction target	Performance
Ahmadi et al.^39^	GA-FL, GA-LSSVM	DT, DEN, CNL, PHIT	$\varnothing$ k	${R}^{2}=0.971$ ${R}^{2}=0.994$
Jamshidian et al.^40^	ANN-ICA	DT, NPHI, RHOB, log(RT), log(K)	$\varnothing$ k	${R}^{2}=0.913$ ${R}^{2}=0.897$
Ahmadi and Chen^41^	LSSVM, FDT, ANN, HGAPSO-LSSVM	DT, NPHI, RHOB, PHIT, and the matched permeability and porosity in a core laboratory (RCAL)	$\varnothing$ k	${R}^{2}=0.975$ ${R}^{2}=0.996$
Zhong et al.^42^	CNN	Gamma ray (GR), bulk density (DEN), the slopes of the GR and DEN curves, and shale content	k	${R}^{2}=0.923$
Zhang et al.^43^	PSO-SVM	GR, DEN, DT, and their slopes	k	${R}^{2}=0.838$
Menke et al.^44^	DBS-Decision Tree	Micro-CT images of estaillades limestone	k	$RMSE=0.43$
Topor^45^	RF	Grain density, core porosity and permeability	k	${R}^{2}=0.834$

## Methodology

**Figure 1:**
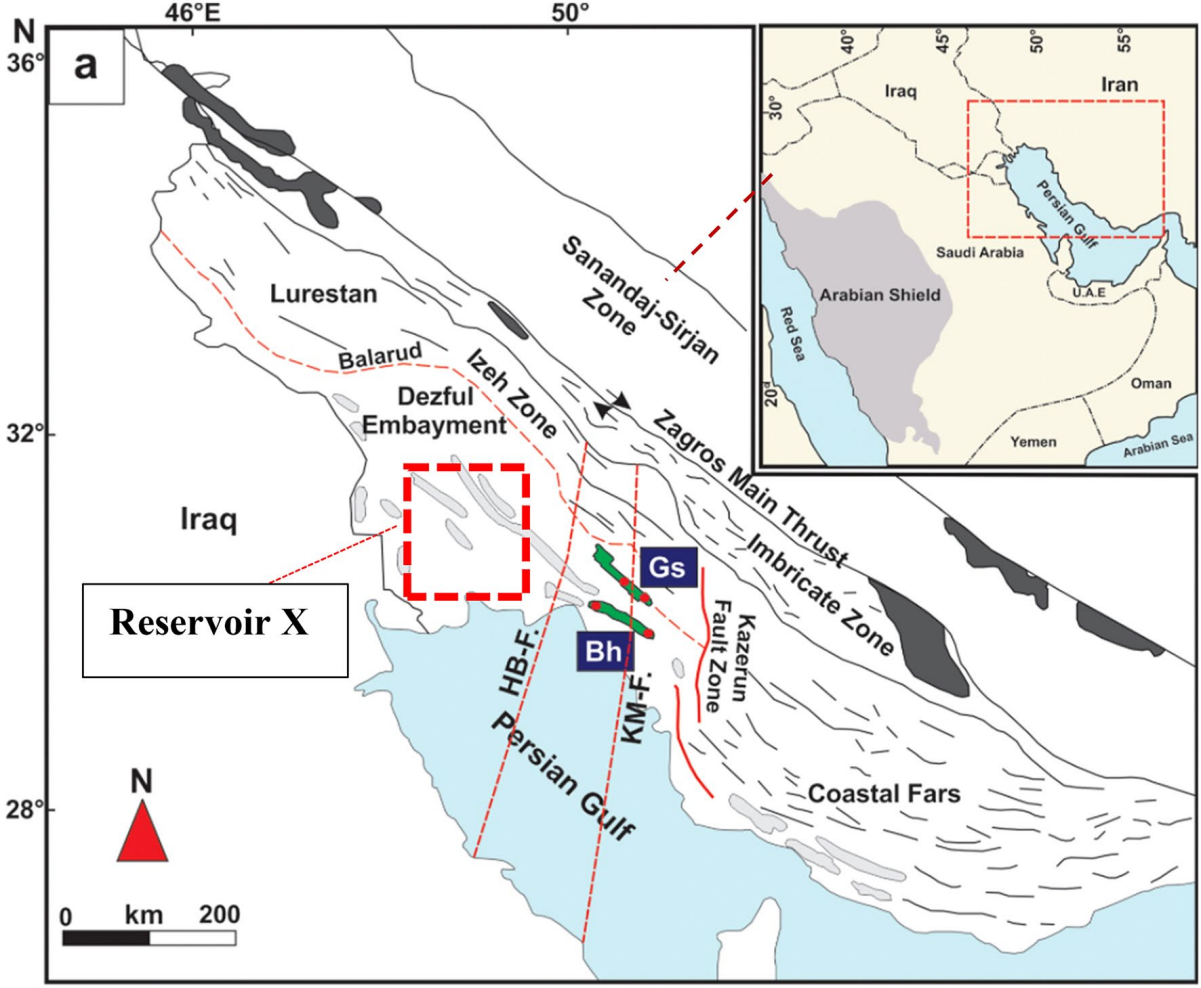
Location of reservoir X, a carbonate reservoir in Asmari formation located in Zagros basin, SW of Iran^46^.

The core data used in this study is obtained from reservoir X, a heterogeneous carbonate reservoir belonging to the Asmari Formation in the Ahvaz field located in the Zagros basin on the northern side of the Persian Gulf, southwestern Iran. Ahvaz field is one of the world’s largest oil fields, and the largest oil field in Iran, and more than 600 wells have been drilled to date. The general trend of the Ahvaz oil field is northwest-southeast resulted from the upward movement of Zagros main thrust-located in the Iranian plateau caused by the collision between Arabian and Indian plates. The main reservoir formations have been deposited during the Oligocene–Miocene and generated a production area of around 67 km long and 6 km wide. All 128 core plugs used in this study were taken at the regular half to one-foot intervals along the zones of interest. Capillary pressure measurements using oil–water centrifuge techniques and mercury injection capillary pressure were conducted on plugs. Table 3 summarizes the statistical background of the database (Fig. 1).

**Table 3:** Statistical description of the database.

	Φ	k(md)	S_wc_	R_35_
Count	128	128	128	128
Mean	0.15	8.87	0.22	1.34
std	0.05	19.08	0.11	1.28
Min	0.05	0.11	0.03	0.25
0.25	0.11	1.07	0.15	0.59
0.5	0.16	2.08	0.21	0.86
0.75	0.18	8.16	0.25	1.55
Max	0.26	122.67	0.77	7.64

In most of the previous studies, FZI is only dependent upon φ, K. However, since FZI is used for petrophysical rock type classification, effects of parameters such as pore throat size and connate water saturation come into play. Hence the modified FZI used in this study (FZIM*) can be defined as:

5 $$ {\text{FZIM}}* = f(\varphi ,{\text{ K}},{\text{ S}}_{{{\text{wc}}}} {\text{R}}_{{{35}}} ). $$

**Figure 2:**
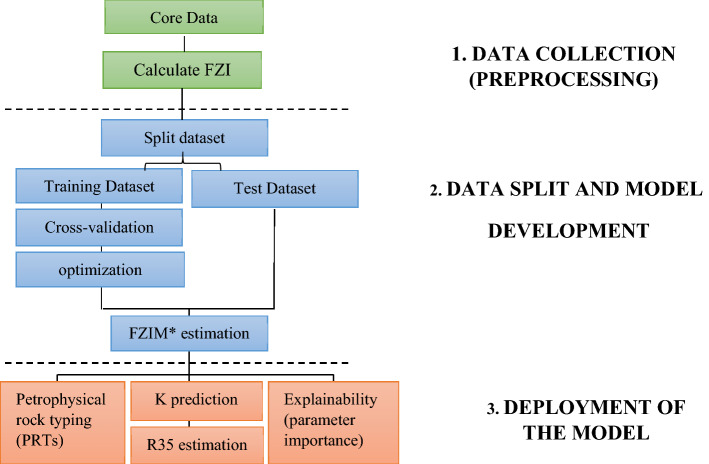
The workflow of the process used in the current study.

**Figure 3:**
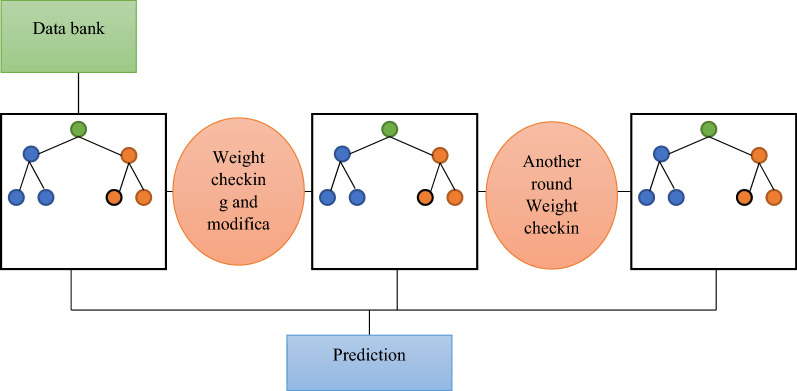
the schematic of a typical griadient boosting algorithm^51^.

The database used in this study was created using φ (fraction), K(md), connate water saturation (S_wc_) in fraction, and R_35_ in microns as input parameters and FZIM* as output. Figure 2 illustrates the overall workflow of the process. The core data was used to train the model without any pre-processing or manipulation, i.e., removing outliers, standardization or normalization of the original data. The FZI to train our model using Eqs. (1)–(4) (Amufele equation^20^) was calculated using φ and K obtained from the core plugs and was fed to the model as the output. Next, the data was split into training (75% of the data) and test datasets (25%). The model used in this study is a type of gradient boosting algorithm coupled with the Bayesian optimization technique and is called Extreme Gradient Boosting (XGB). Model parameters used in this study are summarized in Table 4. The model is implemented using a scikit™ learn package^47^ run in python and is well-known for its speed and performance. The mathematical background of the model is comprehensively explained in work of Chen 2016^48^. A typical schematic of gradient boosting algorithms such as XGB is shown on Fig. 3.

**Table 4:** Model parameters used in this study.

Booster = 'dart'	Early_stopping_rounds = 5
Gamma = 0.0	Learning_rate = 0.2
n-estimatores = 1000	Objective = 'reg:squarederror'
Random_state = 123	CROSS validation accuracy = 0.93

To ensure the model's performance is neither accidental nor due to overfitting of the training dataset, cross-validation process was conducted. Cross-validation is a method to test model validation to assess how statistical analysis results can be generalized to an independent data set. One round of cross-validation typically involves partitioning a sample of data into subsets, performing the analysis on one subset (training set), and validating the results on the other subset (testing set). Next, the same procedure is repeated to another subset; the analysis on each subset is run, and the error/accuracy of all rounds (iterations) is averaged and reported as the cross-validation score. In this study 75% of data is used as a training set, and the remaining 25% was utilized as a testing dataset (fourfold cross-validation).

An optimization process was conducted once the cross-validation results verified the model’s performance. The performance of data-driven/ML methods could be substantially enhanced using a proper optimization process. Various optimization techniques are available, including genetic algorithms, random grid search, and Bayesian optimization^49^. This study applied Bayesian optimization due to its structured nature, efficiency, and speed. It involves a structured method that accelerates finding global optimization problems. It builds a probabilistic model of the objective function that is then searched efficiently with an acquisition function before candidate samples are chosen to evaluate the real objective function. A full description and mathematical background of this method can be found in the work of Snoek et al.^50^. In the next step, the FZIM* was estimated using the testing data set, and the performance of the model was investigated using different model metrics, including r-squared (R^2^), relative error (R.E.), and mean absolute error (MAE) as follows:

6 $$\mathrm{RE}\hspace{0.17em}=\hspace{0.17em}\left|\frac{{x}_{act.}-{x}_{pred.}}{{x}_{act.}}\right|,$$

7 $${\mathrm{R}}^{2}\hspace{0.17em}=\hspace{0.17em}1-\frac{{{\sum }_{i=1}^{N}\left({x}_{i}^{act.}-{x}_{i}^{pred.}\right)}^{2}}{{{\sum }_{i=1}^{N}\left({x}_{i}^{act.}-{\widehat{x}}_{i}^{pred.}\right)}^{2}},$$

8 $$\mathrm{MAE}\hspace{0.17em}=\hspace{0.17em}\frac{1}{n}{\sum }_{i}^{N}\left|{x}_{i}^{act.}-{x}_{i}^{pred.}\right|,$$

where ${x}_{i}^{act.}$ represents FZI and ${x}_{i}^{pred.}$ expresses FZI calculated from the model (FZIM*). One common issue with most data-driven methods previously applied in the industry is their black-box nature and unexplainability. Therefore, the model performance is explained through two analysis: parameter importance and shapely values. These techniques improve the model's explainability through qualitative investigation of the effect of each parameter on FZIM*. Lastly, from the model predicted FZIM*, petrophysical rock types (PRTs), i.e., zones of similar fluid transport properties, were identified. Moreover, permeability and r35 values were estimated using the model output. The estimated permeability and R35 values are essential for parts of the formation where (physical) core samples are not available. Lastly, models performance was explained using two different feature importance analysis (F-score and Shapely), and each feature's effect on the model output was elaborated.

## Result and discussions

**Figure 4:**
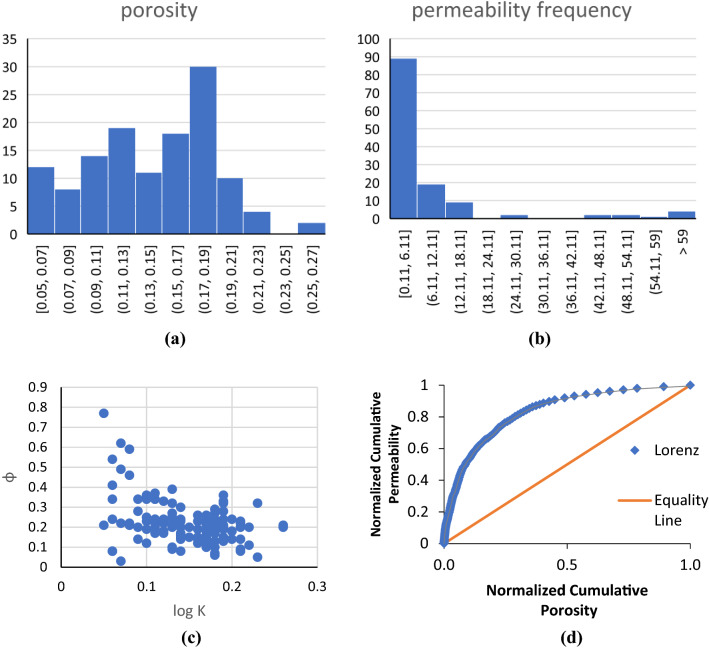
Porosity frequency (**a**), permeability frequency (**b**), porosity vs permeability plot (**c**), Lorenz plot (**d**).

Figure 4 illustrates Poro-permeability, Lorenz plot, and frequency plots of porosity and permeability for the core data used in this study. It can be seen from porosity–permeability that no significant correlation between the two parameters can be observed. The Lorenz Coefficients (LC) of 0.66 further confirms the heterogeneous nature of the reservoir X. Porosity of the reservoir ranges from 0.11 to 0.26; The median for porosity is 0.17, and the majority of the data falls in the range of 0.15–0.19. From the combination of statistical description of permeability in Table 3 and frequency plot of Fig. 4b, more than 90% of the data have permeability lower than 10 md; moreover, the majority of the samples have permeability lower than 6.20 md. Hence, it can be concluded that reservoir X is a heterogenous, low permeability reservoir. In low permeability heterogeneous reservoirs specifically, petrophysical rock typing and permeability prediction is more challenging^52,53^. Therefore, an accurate estimation of PRTs and permeability is more crucial.

### FZIM* prediction using XGB algorithms

**Figure 5:**
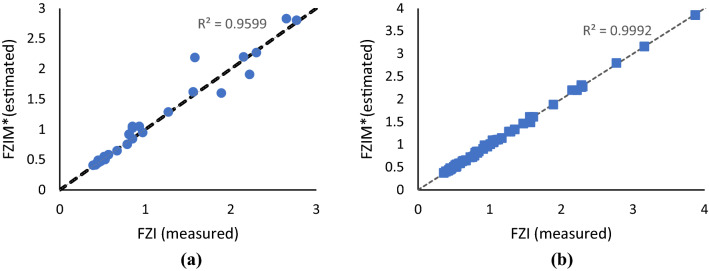
Predicted (FZIM*) versus measured FZI values for Test (**a**) and Training (**b**) datasets.

XGB model accurately predicted FZIM* values (R_2_ > 0.97). Figure 5 shows the cross plot of FZI values measured using core plugs (FZI measured) versus those predicted from the model FZIM* (FZI estimated) for the training and test dataset. The dotted black line represents the unity line (R^2^ = 1). Hence, it can be observed that the model accurately predicts FZI in almost all data points. Moreover, a summary of the model performance (model metrics) is shown in Table 5. It should be noted that the model metrics (MAE, MRE, R^2^) generated from the test dataset are the ones that are of importance as they are based on the data points that the model had not seen previously. The inclusion of the training dataset in the results is merely to provide more insight into model performance. The high cross-validation in the training dataset proves that regardless of the chunk of the original dataset chosen to train the model, the model's performance is still accurate. In other words, high R^2^ scores of cross-validation indicate that the model performance is neither due to the overfitting nor random.

**Table 5:** Summary of model performance in Training and Test Dataset.

Dataset	R^2^	MAE	RE_Max_ (%)	RE_MIN_ (%)	RE_Mean_ (%)	Mean cross validation
Test	0.96	0.08	38.45	0.04	6.89	N/A
Training	0.99	0.01	7.00	0.01	1.84	0.95

**Figure 6:**
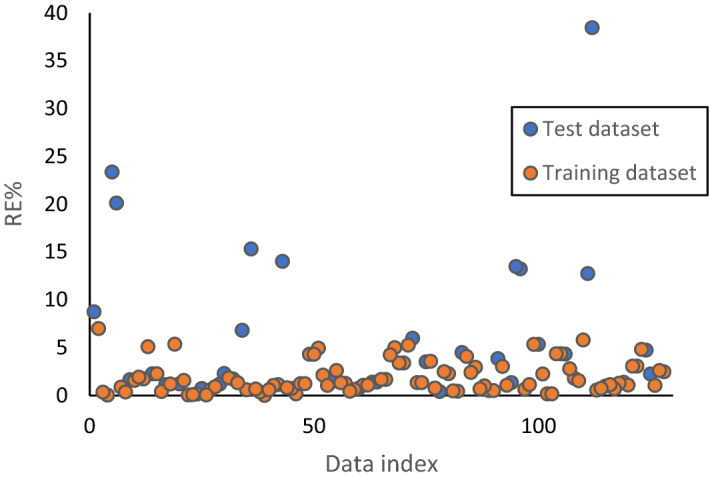
FZI relative error (in percentage) in test and training datasets.

Relative error (in percentage) of predicted FZI (FZIM*) in the training and test dataset is shown in Fig. 6. It can be seen in the figure that with the exception of one data point, all datapoints have a relative error of less than 25%, and the majority of the data both in training and test dataset has relative error lower than 10%. The low relative error indicates that the model performed well in predicting FZI.

### Permeability and R_35_ prediction in uncored sections of the formation

Log data is usually readily available for all the wells. However, the permeability of rock can only be directly measured using cores, or if available, using well-testing methods. Using FZIM*, the permeability in uncored parts of the formation, or from correlated wells for which porosity data is available can be determined. This can be achieved by rearranging Eq. (5) for permeability:

9 $$K=1014 \varnothing {{FZIM}^{*}}^{2}.$$

**Figure 7:**
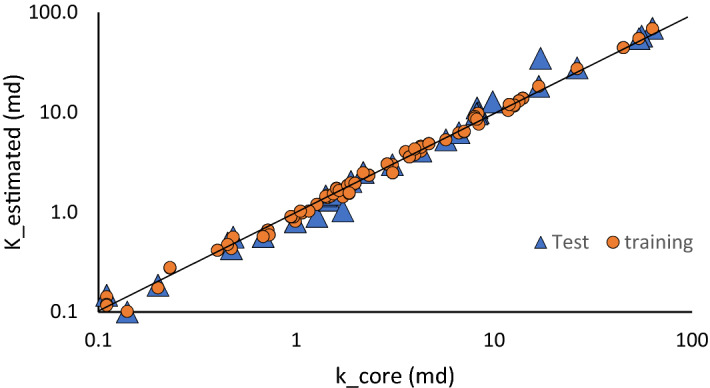
Estimated versus measured core permeabilities for training and test dataset.

Using the estimated FZIM* from the model and porosity values from the core (or log if available), the permeability can be accurately estimated. Figure 7 shows the predicted versus measured permeability values. To better show the dispersion of the values, logarithmic values are used on both axes as the superposition of most of the permeability values is between 0.12 and 10 md. R^2^ between the estimated and actual permeability values for the test and training dataset are 0.97 and 0.99, respectively. It therefore can be seen that using this method, permeability can be accurately predicted.

### R_35_ estimation

Once the permeability is calculated for uncored sections of the well or well-correlated formations of the same reservoir, R_35_ can be calculated from Winland R_35_ equation^18^:

10 $$ {\text{Log R}}_{{{35}}} = \, - 0.{996} + 0.{\text{588 log}}\left( {\text{K}} \right) \, {-} \, 0.{\text{864 log }}\left( \varphi \right). $$

**Figure 8:**
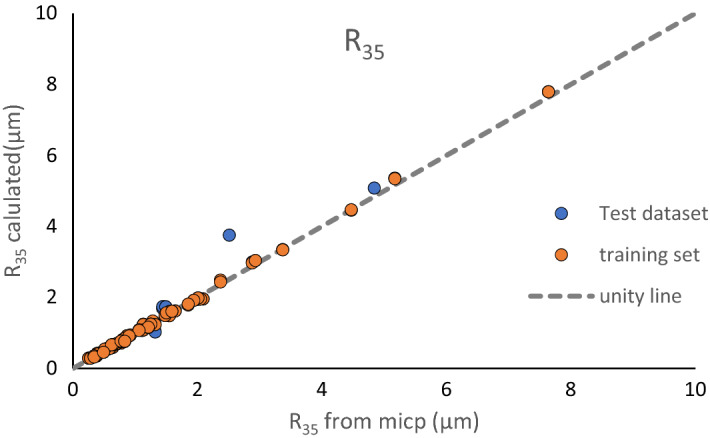
Cross plot of estimated versus measured R35 values for training and test datasets.

The estimated values versus actual values of R_35_ are shown in Fig. 8. R^2^ between the actual and estimated R_35_ values is 0.98 and 0.99 for the test and training dataset, respectively. It can be seen that R_35_ values can be accurately estimated using a combination of FZIM* and Winland equation. This method of calculation of R_35_ could be of significance for dynamic rock typing using Winland equation in sections of well or parts of the formation for which SCAL data such as mercury injection capillary pressure (MICP) and permeability values are not available. There is a direct correlation between pore size and R_35_. Generally, the smaller the pore throat, the lower values of R_35_ and consequently higher capillary pressure (Pc). In low permeability formation, the Winland equation often fails to correctly classify the rock types^54,55^. However, it is still applicable to estimate pore throat radius in each geological zone and petrophysical rock type due to the linear relation between pore throat radius and R_35_ as follows:

11 $${r}_{th}={C}_{1}{R}_{35},$$

where C_1_ is a function of the grain sorting degree in the rock sample^56^.

### Petrophysical rock typing

**Figure 9:**
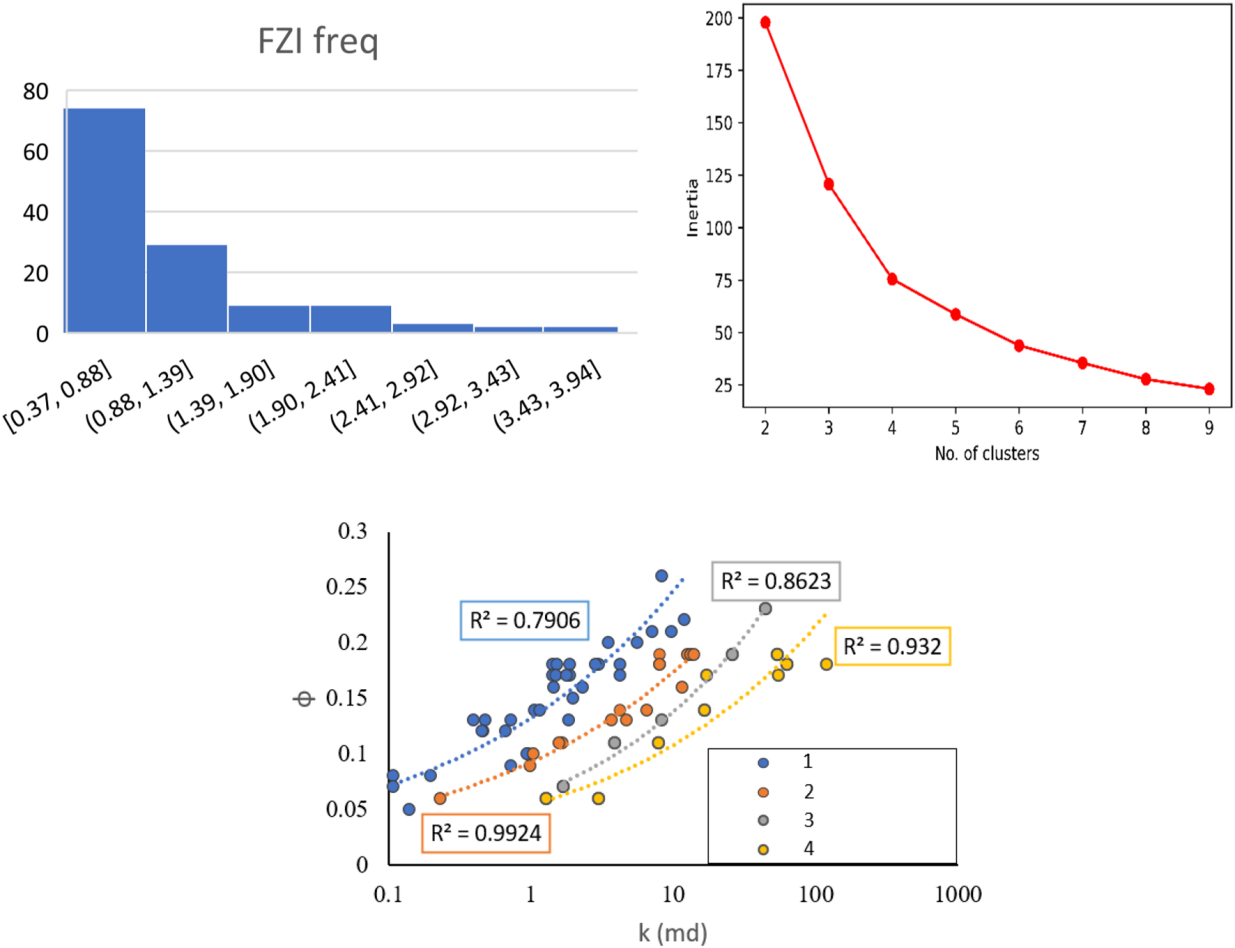
PRT classification based on combination of Frequency and K-means algorithm.

Rocks with similar FZI values can be grouped into a single petrophysical rock type or dynamic rock types^57^. DRT equations and frequency plots are frequently used to cluster the units based on FZI-RQI plot^58^. However, none of these methods alone is sufficient to find the optimum number of flow zones or to efficiently define the boundaries of each zone^59^. To overcome these limitations, an unsupervised machine learning algorithm (K-means clustering method) is used with a frequency plot to find the optimum number of clusters. Clustering analysis initializes by setting the number of clusters to the number of samples, then gradually integrating the samples with similar FZIM* values into joint clusters. To do this, a parameter called inertia is calculated as each cluster is created. The optimum number of clusters could be specified when forming a new cluster, while not substantially reducing the inertia. As shown in Fig. 9, adding more clusters beyond 4 did not lower the inertia substantially. Hence, four PRTs were identified as the optimum number of clusters. Based on Frequency alone, six units were required. However, the combination of K-means and frequency plot resulted in a better PRT clustering compared to using frequency plot alone.

**Figure 10:**
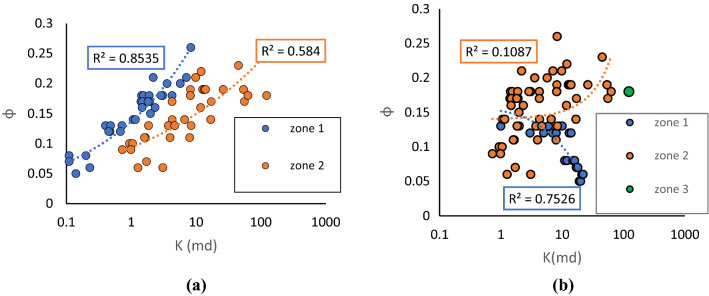
PRT classification based on (**a**) on FZI and (**b**) FZI*.

To compare the performance of the method used in this study, PRT based on two other indices was conducted. Figure 10a,b illustrate petrophysical rock typing based on the well-known FZI^20^ and newly developed FZI*^15,16^. FZI method classifies the reservoir into 2 PRTs while FZI* results in 3 PRTs. PRT based on the FZI approach was more accurate compared to FZI*. However, it can be observed that the R^2^ of PRTs in both scenarios is considerably lower than PRTs using a combination of K-means and FZIM* developed in this study. Hence, the technique developed in the current study was able to classify the PRTs in the heterogeneous reservoir of interest more accurately.

**Figure 11:**
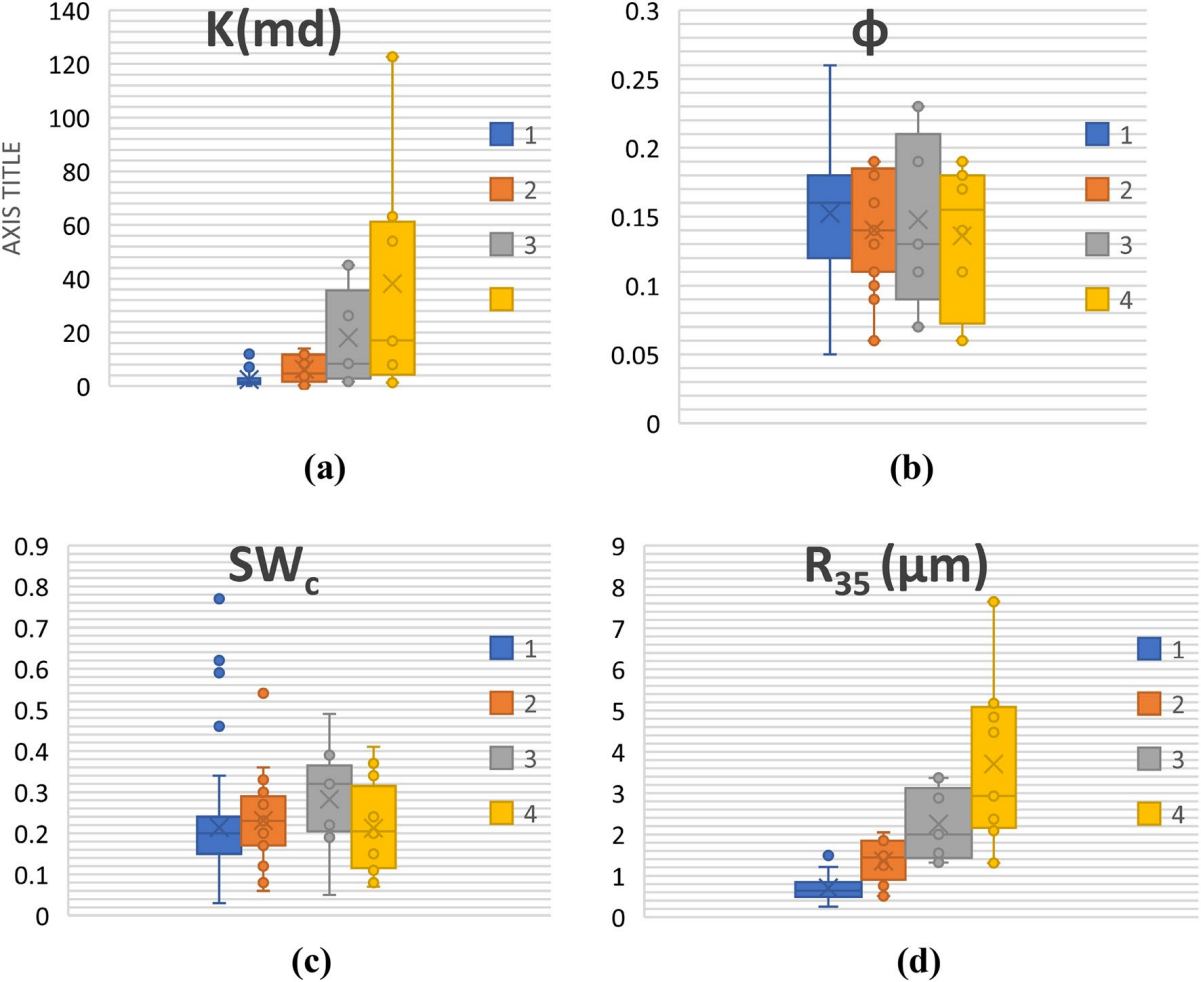
Box plots showing distribution φ, K, Swc and R_35_ for PRTs 1–4.

Cross-plots φ vs. log (K) based for each PRTs estimated using the abovementioned method are shown in Fig. 9. It can be observed that φ versus Log (K) shows a high coefficient of determination across all the zones (0.80 < R^2^ < 0.99). The highest R^2^ can be observed in the second PRT (R^2^ = 0.99), and the lowest is seen in the second zone (0.80). Table 6 summarizes the range of parameters for each induvial PRT. Moreover, the distribution of values for each input parameter (K,φ, S_wc_ and R_35_) is presented in a series of box plots for each PRT (Fig. 11).

**Table 6:** The permeability equations, r-squared (R^2^), and range of FZIM* values, for each PRT**.**

Zones	FZIM* Range	Correlation	R^2^
1	< 0.88	k = 0.755φ^3.714^	0.79
2	0.88–1.39	k = 10.764φ^3.685^	0.93
3	1.39–1.90	k = 16.103φ^2.880^	0.99
4	1.90 >	k = 1.326φ^3.338^	0.86

PRT 4 (yellow color) is characterized by the highest average porosity (up to 13.6%) and permeability (up to 38.1 md), and R_35_ values. This unit has the best storage and flow capacities. PRT 3 (grey color), with an average permeability of 18.3 md and an average porosity of 14.7, is still potentially a good quality zone. PRT 2 (green color), with average permeability of 6.36 and similar values of φ as PRT 3, is a lower quality reservoir. Finally, PRT 1, with an average permeability of 2.45 md and porosity similar to PRT2 and PRT3, is the zone with the lowest flow potential in reservoir X and hence is unlikely to contribute in the production. It can also be observed that the trends seen in R_35_ (Fig. 11d) and permeability (Fig. 11a) are similar. This is primarily due to the relationship between pore throat and permeability. The bigger the pore throat (at 35% of mercury injection in this case), the higher is the permeability of that specific cluster (zone). It should be mentioned that usually, in practice, a hybrid combination of clustering based on lithofacies and electrofacies and FZI is often as the best approach for clustering rather than relying upon one^60^. However, in the current study we tried to investigate clustering methods and parameter estimations (K, R_35_) by only relying on core-driven data^61^.

### Parameter importance

The majority of machine learning methods applications in the literature are of a black-box nature. This means the correlation between each parameter and the output is unclear. Explainable machine-learning is gaining more attention nowadays^30^. To enhance the explainability of the model, two methods of parameter importance analysis, namely F-score and shapely analysis, were conducted. The feature importance using F-score can identify the most influential feature on the output. It estimated the extent to which the model's accuracy is reduced when omitted a specific feature(input). Hence, the input with the highest impact can be identified using this method. On the other hand, Shapley analysis can quantify how different values of inputs positively or negatively affect the output.

**Figure 12:**
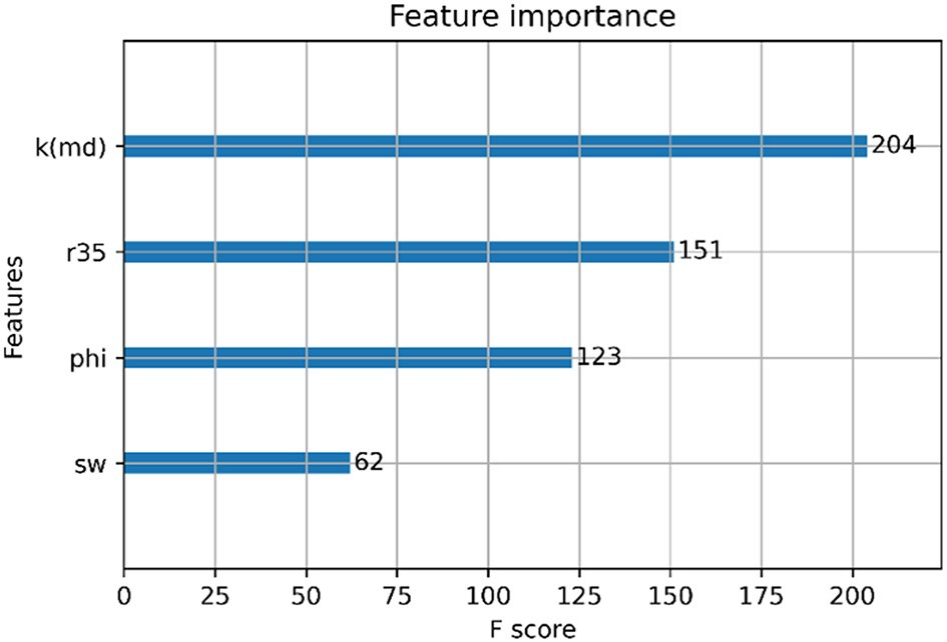
Feature importance of XGB model.

**Figure 13:**
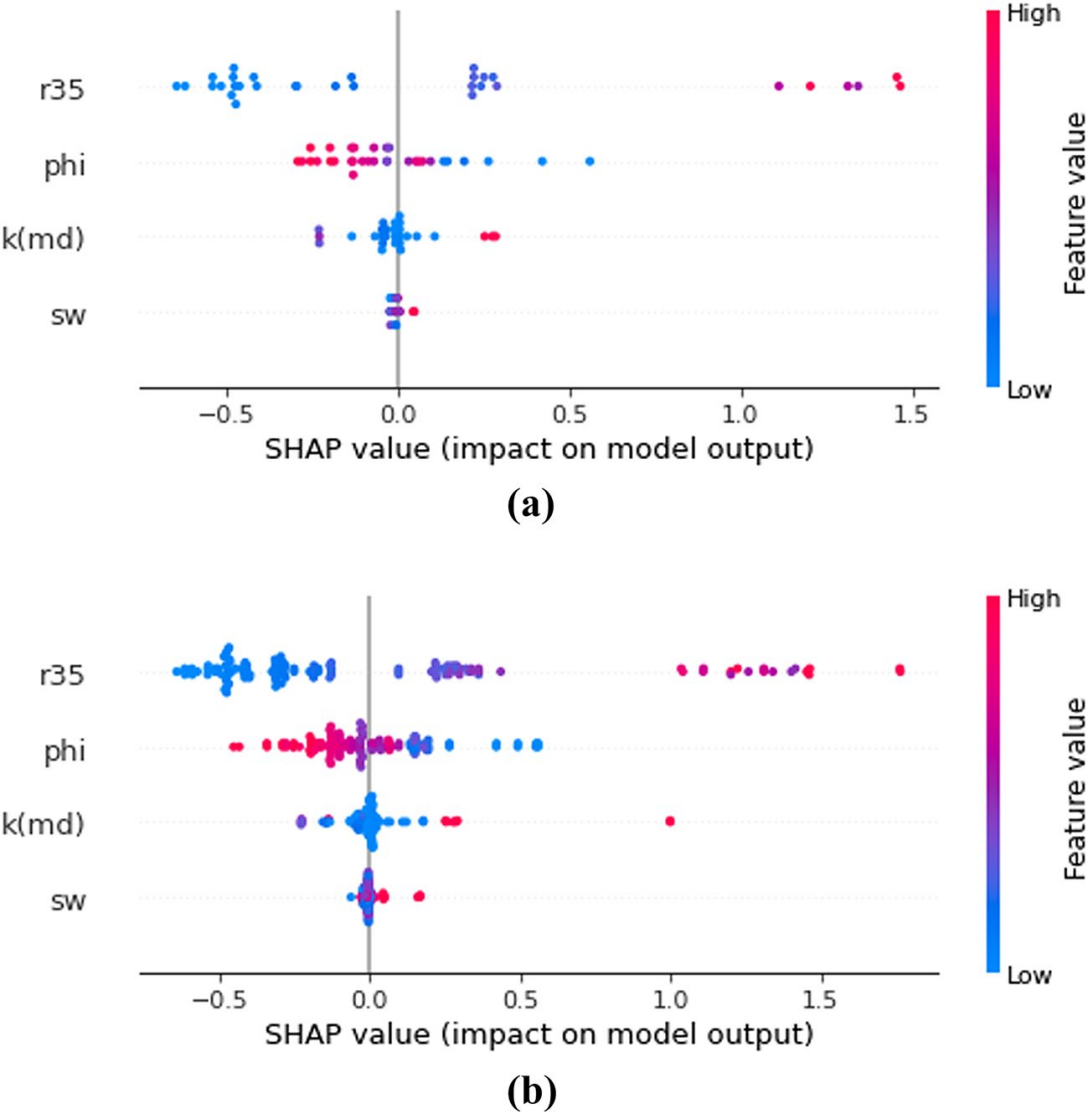
Shapely analysis for Test (**a**) and test + training (**b**) datasets.

Figure 12 shows feature import analysis for the model used in this study. In Fig. 12, the most important feature is permeability, followed by R_35_ and the least important one is S_wc_. Figure 13 shows the shapely values for the testing dataset and full dataset (combination of training and test datasets). First, based on the shapely analysis, it can be seen that R_35_ is the most influential feature where higher values of R_35_ result in higher model outputs (FZI). This is also explainable from the point of view of petroleum engineering as R_35_ corresponds to pore throat radius at 35% mercury injection. Both R35 and k are functions of the pore size. The formation that has larger pores will have larger R35 values, as well as permeability values. Hence, both of the properties mentioned earlier positively impact the model output. The same trends can be observed for permeability (K) and S_wc_ (connate water saturation).

In the case of φ, as observed from Fig. 12, higher porosity values reduce the output. This can be explained through theories behind various FZI indices, as shown in Table 1. FZI represents the productivity/deliverability of a rock type (formation). Hence, for a given porosity, higher permeabilities result in better quality (more productive) zones. As shown in Table 1, the most commonly used FZI values are a function of K/φ. Hence, increasing the porosity results in decreasing the FZI.

Another point worth mentioning is that the same trends of Shapley values can be seen both for Fig. 13a,b. This indicates that the testing dataset (almost being blindly split from the original dataset) is fully representative of the whole dataset. The main advantage of Shapely over the feature importance analysis methods is that the former is model-agnostic. Shapely interpretation utilized game theory's SHAP values to estimate the extent to which each feature contributed to the prediction. The model-agnostic approach consists of using ML/AI models to study underlying structures without assuming that they can be accurately described by the model because of their nature. This reduces potential bias in interpretations^62^.

## Conclusions

In this study, a modified formation zone index (FZIM*) was proposed that in addition to porosity and permeability, is a function of S_wc_ and R_35_.

- A supervised data-driven method (XGB) was used to train the model using the aforementioned parameters and to estimate the FZIM* as an output. The model predictions show a high correlation with the measured FZI values from the core data (R^2^ = 0.97, MAE = 0.08).
- The predicted FZIM* values were then used to calculate permeability and R_35,_ and the estimated values were compared to the measured in both cases high R^2^ between the predicted and estimated parameters was found. Estimated FZIM* values were utilized to create a frequency plot that was then used together with the K-means algorithm to find the optimum number of PRTs. Boxplots of parameters then were used to discuss ranges of parameters in each cluster.
- Lastly, parameter importance and shapely analysis was used to quantify the effect of each input parameter on the output. The novel methodology used in this study (combination of supervised and unsupervised machine learning) can be used to improve petrophysical rock typing and enhance the estimation of parameters such as permeability and pore throat radius that otherwise require expensive cores or well testing.

The current study was conducted using a limited number of core data collected from a heterogeneous carbonate reservoir. The study could be further improved by applying this method to larger datasets. Moreover, including petrophysical parameters such as the cementation factor in the model, could potentially result in an optimized permeability prediction and improved petrophysical rock typing.

## Supplementary Information

Supplementary Information.

## Data Availability

The database and the python code used in the current study are available in Supplementary Information.

## Abbreviations

FZI: Flow zone indicator
MFZI: Modified flow zone indicator
FZIM: Modified flow zone indicator
FZIM*: Modified flow zone indicator developed in this study
HFU: Hydraulic flow unit
PRT: Petrophysical rock typing
PDRT: Petrophysical dynamic rock typing
PSRT: Petrophysical static rock typing
LC: Lorenz coefficient
R^2^: Regression coefficient
RMSE: Root mean square error
SCAL: Special core analysis
RCAL: Routine core analysis
AI: Artificial intelligence
ML: Machine learning
MICP: Mercury injection capillary pressure
XGB: Extreme gradient boosting
RE: Relative error
MAE: Mean absolute error
R_35_: Pore throat radius at mercury saturation of 35%
DRT: Discrete (dynamic) rock typing
RQI: Reservoir quality index
TEM: True effective mobility

## List of symbols

$$\varnothing $$: Effective porosity
$$K$$: Absolute permeability
P_c_: Capillary pressure
S_w_: Water saturation
$${\varnothing }_{z}$$: Porosity
${r}_{mh}$: Mean hydraulic unit radius
F_s_: Shape factor
$$\tau $$: Hydraulic tortuosity
L_a_: Actual length
L: Straight length
S_wc_: Connate water saturation
$\mu$: Fluid dynamic viscosity
$${K}_{r}$$: Relative permeability
r_th_: Pore throat radius
